# Characterization of a pathway−specific activator of edeine biosynthesis and improved edeine production by its overexpression in *Brevibacillus brevis*

**DOI:** 10.3389/fpls.2022.1022476

**Published:** 2022-10-25

**Authors:** Jie Du, Cuiyang Zhang, Qingshan Long, Liang Zhang, Wu Chen, Qingshu Liu

**Affiliations:** ^1^ Hunan Provincial Engineering and Technology Research Center for Agricultural Microbiology Application, Hunan Institute of Microbiology, Changsha, China; ^2^ College of Plant Protection, Hunan Agricultural University, Changsha, China

**Keywords:** *Brevibacillus brevis*, edeines, ParB protein family, EdeB, gene overexpression

## Abstract

Edeines are a group of non-ribosomal antibacterial peptides produced by *Brevibacillus brevis*. Due to the significant antibacterial properties of edeines, increasing edeine yield is of great interest in biomedical research. Herein, we identified that EdeB, a member of the ParB protein family, significantly improved edeine production in *B. brevis*. First, overexpression of *edeB* in *B. brevis* X23 increased edeine production by 92.27%. Second, *in vitro* bacteriostasis experiment showed that *edeB*-deletion mutant exhibited less antibacterial activity. Third, RT-qPCR assay demonstrated that the expression of *edeA*, *edeQ*, and *edeK*, which are key components of the edeine biosynthesis pathway, in *edeB*-deletion mutant X23(ΔedeB) was significantly lower than that in wild-type *B. brevis* strain X23. Finally, electrophoretic mobility shift assay (EMSA) showed that EdeB directly bound to the promoter region of the edeine biosynthetic gene cluster (*ede* BGC), suggesting that EdeB improves edeine production through interaction with *ede* BGC in *B. brevis*.

## Introduction

**Figure 1:**
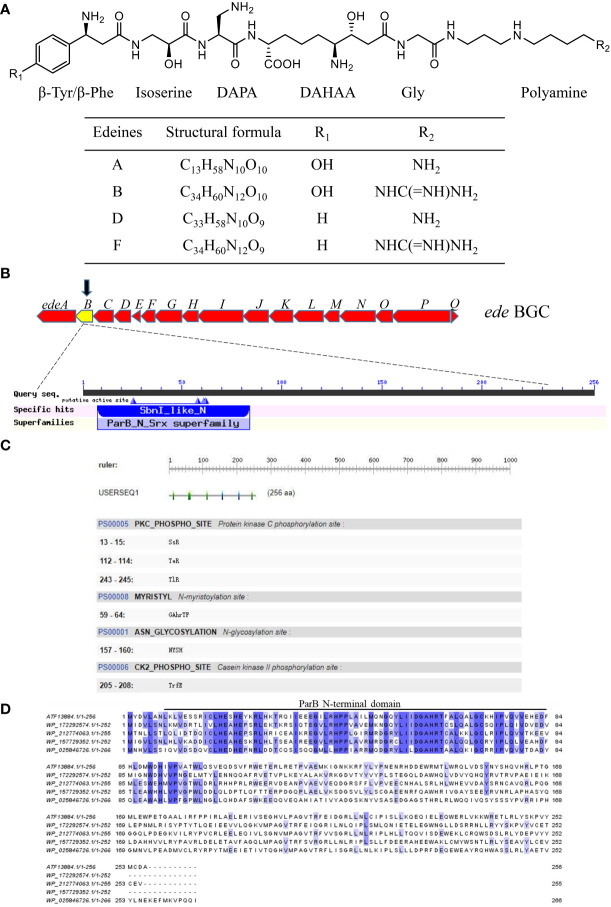
Characteristics of the *edeB* gene and EdeB protein. **(A)** Chemical structure of edeine A, B, D, and F. DAPA, 2,3-diaminopropionic acid; DAHAA, 2,6-diamino-7-hydroxyazaleic acid. **(B)** Bioinformatic analysis of *edeB* gene. The *edeB* gene on the edeines gene cluster (*ede* BGC) was shown in the yellow box. Conserved domain analysis of EdeB protein was performed by NCBI-CDD and Pfam database search. **(C)** Functional sites of EdeB. **(D)** Sequence alignment of EdeB and other ParB family proteins from *Bacillus* sp. *WMMC1349* (GenBank accession: WP_172292574.1), *Polycladomyces abyssicola* (GenBank accession: WP_212774063.1), *Tumebacillus algifaecis* (GenBank accession: WP_157729352.1) and *Brevibacillus agri* (GenBank accession: WP_025846726.1).

*B. brevis* is a Gram-positive, rod-shaped, and spore-producing bacterium, which produces several antibacterial substances, but not toxins (Cook, 1993; Che et al., 2013; Hou et al., 2015; Jianmei et al., 2015). Its antibacterial products are widely used in biological control of plant diseases caused by pathogenic bacteria (Yang and Yousef, 2018; Saxena et al., 2020). Edeines, which are non-ribosomal antibacterial peptides produced by *B. brevis*, consist of four unconventional amino acid residues, a glycine, and a polyamine (**Figure 1A**) (Hettinger and Craig, 1970; Czerwinski et al., 1983; Johnson et al., 2020). As non-ribosomal antibacterial peptides, edeines are synthesized by the multifunctional enzyme complex system of “sulfur template polymerase mechanism”. The unique molecular structure of edeines contributes to a variety of biological function, including antimicrobial and antitumor activities, which can greatly inhibit the growth of numerous bacteria, fungi, mycoplasma, and tumor cells (Kurylo-Borowska and Szer, 1973; Cozzarelli, 1977; Czajgucki et al., 2010). Edeines inhibit bacterial growth through a number of different targets, including DNA, RNA, and ribosomes (protein synthesis), at a dose-dependent manner (Chen and Lu, 2020). At low concentration (<15 μg/mL), edeines reversibly constrain the activity of DNA polymerase II and III to inhibit DNA synthesis, however, the synthesis of protein is not affected. Edeines at high concentration (>150 μg/mL) bind to the P site of small ribosomal subunit (30S subunit), which competitively blocks the binding of fMet-tRNA onto ribosomes and simultaneously inhibits the initiation of transcription and protein translation (Szer and Kurylo-Borowska, 1972; Dinos et al., 2004). In addition, edeines can be used as a transcriptional inhibitor to study ribosome function and protein synthesis. Moreover, edeines can also eliminate bacterial resistance caused by plasmid genes and inhibit the division of *Bacillus subtilis* cells (Shimotohno et al., 2010; Semenchenko et al., 2016).

Regardless of the biological activities of edeines mentioned above, low production level of edeines by edeine-producing bacteria is a major issue limiting their applications in medicine and agriculture. Edeine A and B produced by *B. brevis* X23 exhibit wide antibacterial spectrum and have been used as biocontrol agents against bacterial wilt in solanaceae crops. The complete genomic sequence of *B. brevis* X23 allows the identification of edeine biosynthetic gene cluster (*ede* BGC) (Wu et al., 2012; Liu et al., 2016). In this study, we identified and explored the biological function of regulatory proteins involved in biosynthesis of edeines, which will provide a theoretical basis for the construction of *B. brevis* strains with high-yield of edeines through genetic manipulation.

A potential transcriptional factor gene *edeB* on the *ede* BGC was identified on the basis of bioinformatic analysis of *B. brevis* X23 genome. Protein EdeB contains a typical helix-turn-helix (HTH) domain, which is conserved in ParB family transcriptional factors. Although ParB homolog proteins have been annotated from many genomes of *Bacillus* species, their function has not been fully understood yet. In this study, we constructed a *B. brevis* mutant X23(ΔedeB) by disrupting the *edeB* gene through Red/ET homologous recombination. Furthermore, a complementary strain and *edeB*-overexpression strain were constructed using the expression plasmid PAD-Apr-Px-edeB vector. Subsequently, we compared edeine production among those different *B. brevis* strains to explore the effect of *edeB* on the yield of edeines. The binding ability between EdeB protein and the promoter region of *ede* BGC was also evaluated. Our results demonstrated that EdeB significantly improved edeine production in *B. brevis* X23 through binding to *ede* BGC.

## Materials and methods

### Strains, plasmids, culture medium, and antibiotics

The strains and plasmids used in this study are shown in **Table 1**. *E. coli* and *B. brevis* were cultured in LB medium (tryptone 10.0 g/L, yeast extract 5.0 g/L, NaCl 1.0 g/L, pH 7.0 ~ 7.5, and 1.5% of agar powder for solid culture). NB medium (tryptone 10.0 g/L, beef extract 3.0 g/L, sodium chloride 5.0 g/L, glucose 10.0 g/L, pH 7.0 ~ 7.5) was used for fermentation cultivation. Apramycin (20 μg/mL) and Kanamycin (30 μg/mL) were added into the media for *E. coli* as required. Gene-engineered *B.brevis* strains were selected on LB media containing apramycin (10 μg/mL).

**Table 1:** Strains and plasmids used in this study.

Strains and plasmids	Characteristic	Source
Strains		
*B. brevis* X23	wild-type	Laboratory preservation
*B. brevis* X23(ΔedeB)	Apra^r^, *edeB*-deletion mutant	This study
X23(ΔedeB) (PAD-Apr-Px-edeB)	complementary strain of X23(ΔedeB)	This study
X23 (PAD-Apr-Px-edeB)	*edeB* overexpression strain	This study
*B. subtilis* 1A751	Derivative strain of *B. subtilis* 168	This study
*Escherichia coli* GB-Dir	GB2005, *ara*C-BAD-ETγA	Laboratory preservation
BL21(DE3)		Laboratory preservation
Plasmids		
pSET152	Apra^r^	Laboratory preservation
pBR322-ErmB-pE194	knockout vector	This study
pE194-*edeB*-ko-HAF-apra-HAR	*edeB* gene knockout vector	This study
PDG148-Cre-Erm	Cre (Cyclization Recombination Enzyme) plasmid	Laboratory preservation
PAD-Apr-Px-edeB	*edeB* gene overexpression vector	This study
pET28a-edeB	Recombinant protein expression vector	This study

Apra^r^, apramycin resistance.

### Reagents

PrimerSTAR Max DNA Polymerase and DNA molecular weight standard were purchased from TaKaRa Co., Ltd (Dalian, China). All restriction endonucleases were purchased from New England Biolabs (Beijing, China). Genomic DNA, RNA, and plasmid extraction kits, reverse transcription kits, and DNA gel recovery kits were purchased from TransGen Biotech (Beijing, China). Antibiotics and other biochemical reagents were purchased from Sangon Biotech Co., Ltd. (Shanghai, China).

### Primers, genomic DNA extraction, and DNA sequencing

Primers used in this study are shown in **Supplementary Table 1**. All primers were synthesized by Sangon Biotech Co., Ltd. (Shanghai, China). The genomic DNA of *B. brevis* X23 was extracted using the phenol chloride method according to previous study (Mcorist et al., 2002). DNA sequencing was conducted at Hunan Tsingke Biotechnology Co., Ltd. (Changsha, China).

### Identification of the regulatory gene *edeB* on *ede* BGC

The genome of *B. brevis* strain X23 was sequenced and deposited at NCBI with the accession number NZ_CP023474.1. Regulatory genes around *ede* BGC in *B. brevis* X23 genome were reviewed to identify *edeB*. The conserved domain of identified EdeB protein (Genebank accession: ATF13884.1) was analyzed by the NCBI’s conserved domain database (https://www.ncbi.nlm.nih.gov/cdd/) and Pfam (http://pfam.xfam.org/). The function sites of EdeB were analyzed by PROSITE (https://prosite.expasy.org/).The homologs of EdeB were compared and analyzed using Jalview software.

### Construction of *edeB*-deletion, complementation, and overexpression mutants

The *edeB*-knockout vectors were constructed based on Red/ET homologous recombination (Yu et al., 2000; Zhang et al., 2000; Muyrers et al., 2001; Sharan et al., 2009). Genomic DNA of *B. brevis* X23 was used as a template to amplify the upstream and downstream homologous arms of *edeB* with primers edeB-F1/edeB-F2 and edeB-R1/edeB-R2 using PCR assay. Subsequently, the apramycin resistance gene (Apra^r^) was amplified from plasmid pSET152 with primers Apra-F/Apra-R using PCR. The pBR322-ErmB-ori194 vector skeleton was amplified plasmid pE194 with primers pE194-F/pE194-R. Then, the above four DNA fragments were mixed and electroshocked into *E. coli* GB-Dir containing the Red/ET recombination system to be connected into a circular plasmid through homologous recombination. Then proper knockout vectors were selected and sequenced through enzyme digestion and the selected plasmid with correct sequence was named as pE194-edeB-ko-HAF-apra-HAR. The knockout vector was transferred into *B. brevis* X23, and undergone homologous recombination to replace the *edeB* gene by Apra^r^. The mutant was further confirmed through PCR assay with primers P1/P2 and P3/P4, and named as *B. brevis* X23(ΔedeB)-Apra. Finally, the Cre plasmid was transferred into *B. brevis* X23(ΔedeB)-Apra to generate *B. brevis* X23(ΔedeB) strain by removing Apra^r^.

Next, the *edeB* gene was amplified by PCR with primer pairs edeB-Px-F and edeB-Px-R. The amplicons were recovered from agarose gel and purified. The *edeB* gene fragments and vector PAD-Apr-Px were digested with *BamH I* and *Pst I* endonucleases, respectively, and then connected by T4 DNA ligase to obtain the expression plasmid PAD-Apr-Px-edeB. Finally, the expression plasmid was transferred into wild-type *B. brevis* X23 strain and X23(ΔedeB) by electro-transfer. The *edeB*-complementary and overexpression strains were obtained through apramycin antibiotic resistance screening, and confirmed by PCR with primers xylR-5-out/pAD123-end.

### Assessment of bacterial growth

The growth of wild-type *B. brevis* strain and its mutants were evaluated according to the previously published report (Lan et al., 2017). Briefly, the wild-type *B. brevis* X23 strain or mutants were preconditioned overnight in LB medium, and then transferred to fresh NB liquid medium (initial OD_600_ of 0.1), cultured at 30°C and 180 r/min. Then, the bacterial solution concentration (OD_600_) was measured on a spectrophotometer after 12, 24, 36, and 48 h of cultivation in the NB liquid medium. The fermentation experiments were repeated for three times, and each measurement was also conducted for three times in parallel.

### Evaluation of antibacterial activity

*B. subitlis* 1A751 strain, which is sensitive to edeines, was used to evaluate the antibacterial activity of edeines produced by *B. brevis*. The antibacterial activity of fermentation broth was evaluated according to the previous study (Dang et al., 2013). Briefly, *B. brevis* was cultivated in liquid NB for 48 h, and centrifugated at 10,000 g for 10 min at 4°C to collect the supernatant, which was then filtrated through a filter (0.22 μm) to obtain sterile filtrate. *B. subtilis* 1A751 was spread on LB agar plates after dilution to OD_600_ of 0.1. A total of 100 μL filtrated *B. brevis* supernatant was added into each hole (diameter of 8 mm) of *B. subtilis* culture. Sterile LB medium was used as blank control (CK). After incubation at 30°C for 48 h, the antibacterial activity was evaluated by measuring the radius of antibacterial circle. The experiments were repeated for three times with three replicates each.

### RNA isolation and RT-qPCR

The relatively transcriptional level of *edeA*, *edeQ*, and *edeK* on *ede* BGC was determined through RT-qPCR assay. The primers used on qPCR assay are shown in **Supplementary Table 1**. Total RNA was extracted from *B. brevis* X23 and mutants after 48h of fermentation according to the instructions of EasyPure ^®^ RNA Kit. Reverse transcription was conducted using the reverse transcription kit (EasyScript^®^One-Step gDNA Removal and cDNA Synthesis SuperMix, TransGen). 2X Universal SYBR Green Fast For qPCR Mix kit (Abclone) was used in real-time fluorescent quantitative PCR assay on an ABI-QS5 real-time PCR experiment platform. Three parallel experiments were designed and each sample was repeated three times. The 16S rRNA gene in *B. brevis* was used as a control, and the comparative cycle threshold method was used for relative quantification (Livak and Schmittgen, 2013).

### Analysis of the promoter sequence of *ede BGC*

The binding sites of *ede* BGC promoter were predicted through BPROM (http://linux1.softberry.com/berry.phtml?topic=bprom&group=programs&subgroup=gfingf) and BDGP (https://www.fruitfly.org/seq_tools/promoter.html). WeBlogo 3.0 (http://weblogo.threeplusone.com/) was used to create sequence logos for the -10 and -35 regions of the promoter.

### Electrophoretic mobility shift assay (EMSA)

EMSA was conducted to explore whether EdeB directly binds to the promoter region of *ede* BGC according to the previous method (Hellman and Fried, 2007). Briefly, the *edeB* gene was amplified from genomic DNA of wild-type *B. brevis* X23 using primers edeB-F and edeB-R. The vector pET28a was digested by *Xba I* and *Xho I*, and then incubated with purified amplicons of *edeB*. Then, the mixture was electroshocked into *Escherichia coli* GB05-dir containing the recombinant system through homologous recombination. The extracted plasmid was identified and transformed into competent cells BL21(DE3) to obtain recombinant strain BL21/pET28a-edeB. The EdeB protein was purified from the culture after IPTG induction. The promoter region *edePro* of *ede* BGC was amplified by PCR using primer pairs edePro-F/edePro-R with 6-FAM at the 5’end (**Supplementary Table 1**). Subsequently, the labeled DNA fragments were mixed with purified EdeB protein. The binding reaction system (20 uL) consists of 10 mM Tris (pH 7.5), 5 mM MgCl2, 50 mM EDTA, 60 mM KCl, 10 mM DTT, 10% glycerol, indicated amounts of EdeB protein and purified *edePro* composition. Randomly interrupted salmon sperm DNA was added to the binding reaction system to prevent nonspecific competition. After incubation on ice for 10 minutes, the reactants were run on 8% of TBE polyacrylamide gel (Bio-Rad) with 0.5×TBE running buffer for 1 hour at 30 mA.

### Evaluation of edeine production through HPLC–MS

Edeines produced by wild-type *B. brevis* X23 and its mutants were quantitatively analyzed by HPLC-MS. Briefly, HPLC-MS was performed on the HPLC system (Agilent 1260) in an ODS column (Shimadzu AQ-C18 HP, 2.1 mm×100 mm×3μm) with the following parameters: solvent A, acetonitrile; solvent B, 0.1% formic acid-water; elution procedures:5% A,10 min, 100% A, 2 min; flow rate was 0.3 mL/min; column temperature 40°C. Mass spectrometry conditions: MRM scanning mode, positive ion, molecular weight scanning range of 100 m/z. 1000 m/z, nebulizer flow rate of 3 L/min, heating gas flow rate of 10 L/min, interface temperature of 200°C, DL temperature of 200°C, heating block temperature of 350°C, and dryer flow rate of 10 L/min. The peak areas of edeine A (m/z 755.444 [M+H]^+^) and B (m/z 797.465 [M+H]^+^) were compared by HPLC-MS.

## Results

### *edeB* encodes a putative ParB family protein

The protein EdeB (Genebank accession: ATF13884.1) encoded by the *edeB* gene consists of 256 amino acids with a molecular weight of 30.4 kDa. Bioinformatics analyses showed that EdeB protein was a member of the ParB family proteins, and the N-terminal of EdeB contained a SnbI_like domain (**Figure 1B**). PROSITE analysis identified a number of functional sites in EdeB, including Protein kinase C phosphorylation, N-myristoylation, and N-glycosylation, and Casein kinase II phosphorylation sites (**Figure 1C**). The amino acid sequences of EdeB and three homologs of ParB family proteins from other bacterial species were aligned and reviewed using the Jalview software. The results showed that EdeB was a member of the ParB family proteins with highly conserved sequences (**Figure 1D**).

### Disruption of *edeB* significantly reduced edeine biosynthesis and inhibited the antibacterial activity of *B. brevis* X23

**Figure 2:**
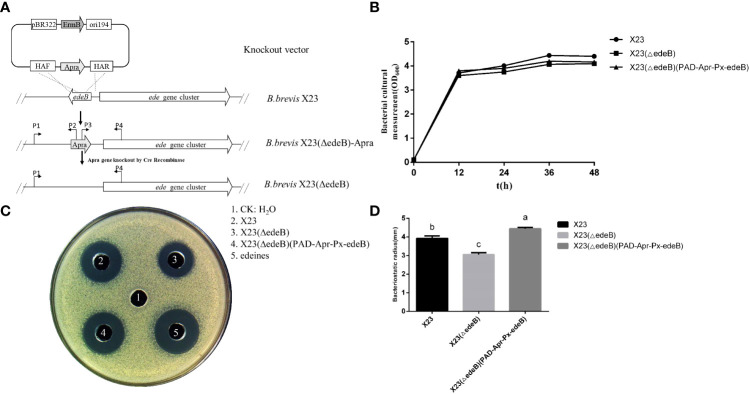
Comparison of antibacterial activity between *edeB* -deletion mutant and complementation strains. **(A)** *edeB* gene knockout flow chart. **(B)** Growth curve comparison. **(C)** Antibacterial ability comparison. **(D)**Comparison of radius of the inhibition zone. Different letters indicate statistically significant differences (*P* < 0.05).

In order to explore the role of *edeB* in edeine biosynthesis, the *edeB* gene was replaced by apramycin resistance gene through Red/ET homologous recombination, and the mutant was named as X23 (ΔedeB)-apra. Then the Cre enzyme plasmid was transferred into X23 (ΔedeB)-apra to finally obtain *B. brevis* X23(ΔedeB) strain through removing the Apra^r^ (**Figure 2A**). We compared the cell growth curves of wild-type strain *B. brevis* X23, *edeB*-deletion mutant X23(ΔedeB), complementary strain X23(ΔedeB)(PAD-Apr-Px-edeB) in NB fermentation medium. The results showed that no significant difference in the cell growth curves was detected among the wild-type *B. brevis* X23, X23 (ΔedeB), and X23(ΔedeB)(PAD-Apr-Px-edeB). (**Figure 2B**).

The antibacterial activity of fermentation products was compared among wild-type *B. brevis* X23, X23 (ΔedeB), and X23(ΔedeB)(PAD-Apr-Px-edeB) cultured in NB medium for 48 hours. The results showed that the inhibition zone of X23 (ΔedeB) was significantly smaller than that of the wild-type *B. brevis* X23. The inhibition zone of the X23 (ΔedeB) (PAD-Apr-Px-edeB) was significantly larger than that of wild-type *B. brevis* X23 (**Figures 2C, D**). Taken together, *edeB* disruption significantly inhibited the antibacterial activity of *B. brevis* X23.

**Figure 3:**
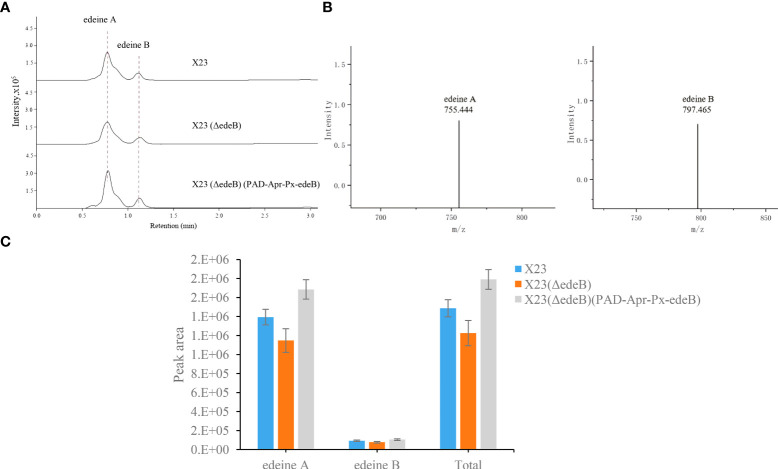
Comparison of edeine production among wild-type *B*. *brevis* X23, X23 (ΔedeB), and X23 (ΔedeB) (PAD-Apr-Px-edeB) by HPLC-MS. **(A)** Comparison of edeine yield by HPLC-MS analysis. The retention time of edeine A and B was indicated by the dotted lines. **(B)** The mass spectrogram of edeine A and B. The molecular ions [M+H]^+^ were 755.444 m/z and 797.465 m/z, respectively. **(C)** Change of edeine A and B yield after 48 h fermentation.

To further explore the effect of EdeB on edeine biosynthesis, HPLC-MS was used to compare edeine production among wild-type *B. brevis* strain X23, X23 (ΔedeB), and X23 (ΔedeB) (PAD-Apr-Px-edeB). The results showed that X23 (ΔedeB) still produced edeines, but the yield of edeine A and B decreased by 17.67% and 16.16%, respectively, compared with wild-type *B. brevis* strain X23. *edeB* gene complementation increased the yields of edeine A and B in X23 (ΔedeB) (PAD-Apr-Px-edeB) by 20.88% and 13.53%, respectively, compared with wild-type *B. brevis* X23 (**Figure 3**). These results confirmed that EdeB improved edeines biosynthesis, which is consistent with the above results of antibacterial activity evaluation.

### Deletion of *edeB* gene significantly inhibited the expression of *ede* BGC

To study the effect of EdeB on the transcription of *ede* BGC, we compared the expression of genes *edeA* (encoding a transporter protein), *edeQ* (encoding a resistance enzyme), and *edeK* (encoding a non-ribosomal peptide synthase, NPRS) between X23(ΔedeB) and wild-type *B. brevis* X23. And the 16S rRNA gene with stable transcriptional expression was selected as the internal reference.

**Figure 4:**
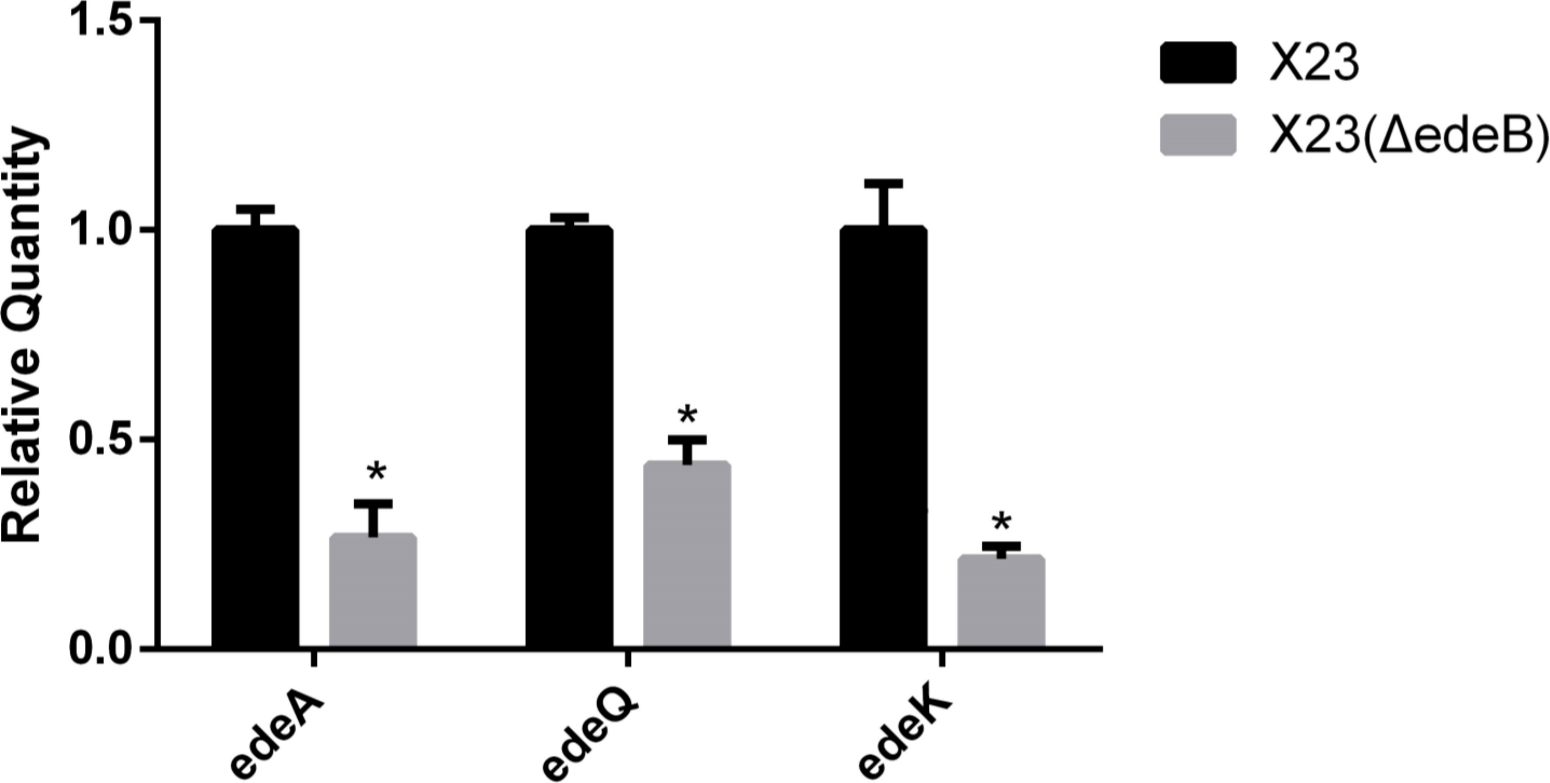
Effects of *edeB* gene knockout on the transcription levels of *edeA*, *edeQ*, and *edeK*. * statistically significant difference.

The value of the target gene measured by the ΔΔCt formula. Greater than 2.0 or less than 0.5 means that the transcription level of the target gene is significantly increased or decreased. The RT-qPCR results showed that the transcription level of *edeA*, *edeQ*, and *edeK* in X23(ΔedeB) significantly decreased compared with that in the wild-type *B. brevis* X23, suggesting that EdeB was a positive regulator in edeine biosynthesis (**Figure 4**).

### Bioinformatics analysis of the main elements of promoter *edepro* in *ede* BGC

**Figure 5:**
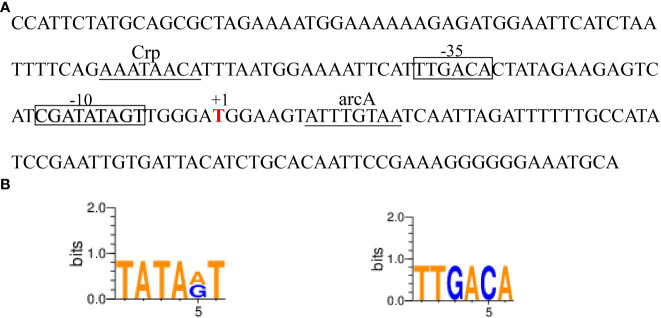
The edeine promoter *edePro*. **(A)** Bioinformatic analysis of *edePro* by BPROM and BDGP. **(B)** Conservative identification of -10 and-35 region in *edePro*.

The *ede* BGC promoter (~200 bp), namely *edePro*, has been previously identified and characterized (Liu et al., 2022). Bioinformatic analyses identified a -10 and -35 region in the sequence of *ede* BGC promoter *edePro* (**Figure 5A**). The transcriptional factor binding site (TFBS) sequences were involved in binding with transcription factors Crp (cAMP receptor protein) and arcA (Arginine deiminase). WebLogo 3.0 conservative identification program showed that the 6-nucleotide sequence in the -10 region was similar to the classical -10 region (TATAAT), and the 6-nucleotide sequence in the -35 region was identical to the classical -35 region (TTGACA) (**Figure 5B**).

### EdeB directly regulates the promoter region of *ede* BGC

**Figure 6:**
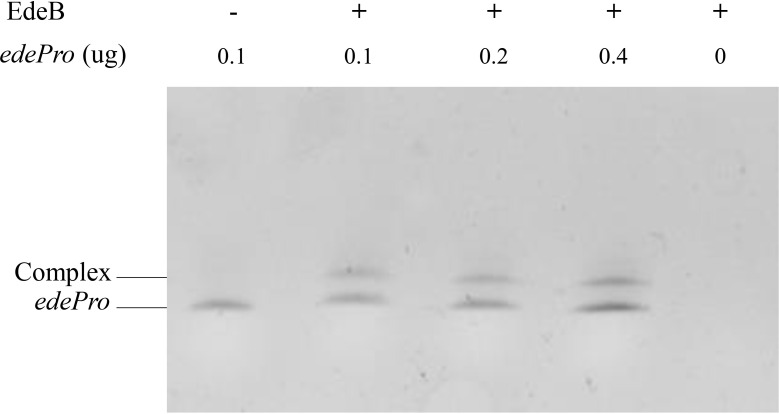
The binding ability of EdeB to ede BGC analyzed by EMSA. – : 0 μg EdeB protein; + : 1 μg EdeB protein.

Transcriptional regulators usually control transcription by specifically binding to the promoter region of target genes (Leslie and Justin, 2013). In order to study whether EdeB regulates edeine biosynthesis through the same way, EMSA was used to evaluate the binding ability of EdeB with the promoter region of *ede* BGC. The results showed that EdeB inhibited the migration of promoter fragment *edePro*, suggesting that EdeB directly interact with *ede* BGC. The interaction between EdeB and *ede* BGC was further confirmed (**Figure 6**). Taken together, EdeB improved the transcription of *ede* BGC and edeine production in *B. brevis* by directly binding to its promoter region.

### *edeB* overexpression enhanced edeine production

**Figure 7:**
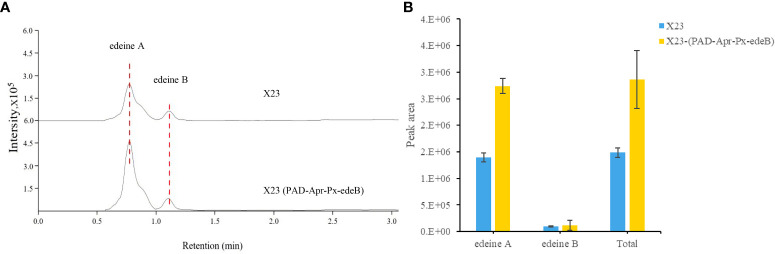
Edeine production in wild-type *B. brevis* X23 and X23 (PAD-Apr-Px-edeB). **(A)** Comparison of edeine yield by HPLC-MS analysis. The retention time of edeine A and B was indicated by the dotted lines. **(B)** Change of edeine A and B yield after 48 h fermentation.

The yield of edeine A and B in X23 (PAD-Apr-Px-edeB) was increased by 96.60% and 27.26%, respectively, by *edeB* gene overexpression and the total yield of edeines was increased by 92.27% (**Figure 7**). These results confirmed that EdeB improved edeine biosynthesis.

## Discussion

Edeines are natural nonribosomal peptides with antibacterial, antifungal, and antitumor activities (Johnson et al., 2020). Edeines are produced by *B. brevis* under appropriate condition, however, only a few reports focusing on the molecular pathway and regulatory factors involved in edeine production in *B. brevis*. Westman first identified the edeine biosynthetic gene cluster in *B. brevis* Vm4, and confirmed that *edeQ* was a resistance gene. Heterologous expression of *edeQ* enhanced resistance of edeines in *Bacillus subtilis* (Westman et al., 2013). In addition, Zhang et al. reported that AbrB downregulated the biosynthesis of edeines in *B. brevis* X23, and *abrB* knockout improved the yield of edeines (Zhang et al., 2020).

Currently, our understanding of the molecular pathway and regulation of edeine biosynthesis is still limited. In this study, we identified that the regulatory gene *edeB* on *ede* BGC was involved in edeine biosynthesis of *B. brevis* X23. Bioinformatics analysis showed that EdeB protein is a member of the ParB family, which are plasmid active distribution proteins involved in the distribution of plasmids and chromosomes, as well as embodying DNA-binding activity (Jalal et al., 2020). Recent studies have shown that many ParB family proteins functioned as transcription factors in numerous biological processes (Khare et al., 2004). In addition, EdeB protein contains a typical HTH domain, which can bind with DNA, suggesting that EdeB protein plays a regulatory role in transcription (Kusiak1 et al., 2011). While ParB family proteins are common in *Bacillus*, their role in the regulation of antibiotic biosynthesis has not been clearly studied (Webb et al., 2010). SbnI, a protein of the ParB superfamily, is a heme-inducible transcriptional regulator involved in Staphyloferrin B (SB) production (Laakso et al., 2016). SbnI also exhibited enzymatic function in SB biosynthesis (Skaar et al., 2004). It has been shown that Glu^20^ and Asp^58^ were required for the kinase activity of SbnI (Verstraete et al., 2018). The N-terminal of EdeB protein contains a SnbI_like domain, suggesting that it may has similar function as SnbI.

The important role of gene *edeB* in the edeine biosynthesis was confirmed by *edeB* knockout and complementation experiments. The inhibition zone of *B. brevis* strain with *edeB* knockout significantly smaller, and the antibacterial activity was significantly reduced compared with the wild-type *B. brevis* strain X23. Gene *edeB* complementation restored and improved its antibacterial ability. HPLC-MS results showed that *edeB* knockout reduced edeine production by 18%, but *edeB* complementation increased edeine production by 20.43%. Taken together, these results suggest that *edeB* was essential for edeine biosynthesis by upregulating edeine production.

The expression of *edeA*, *edeQ*, and *edeK* on *ede* BGC was analyzed by RT-qPCR. The results showed that the expression of these three genes were significantly reduced in *edeB*-deletion mutant. The genes with decreased transcription were involved in antibiotic transport, core skeleton formation, and resistance in edeine biosynthesis. Reduced transcription of these genes may be regulated by EdeB, which is consistent with decreased production of edeines in *edeB*-deletion mutant, suggesting that *edeB* is a positive regulator in the biosynthesis of edeines.

EMSA analysis showed that EdeB could directly bound to the promoter region of *ede* BGC. Based on the direct interaction between EdeB and *edePro*, and *edeB* deletion and complementation experiments discussed above, we propose a molecular mechanism underlying the regulatory role of EdeB in edeine biosynthesis. EdeB upregulates the expression of the genes on *ede* BGC to improve the production of edeines through binding to the promoter region of *ede* BGC. However, whether other proteins are involved in this process or interact with EdeB is still not known. In summary, EdeB is a major component in edeine biosynthesis. Overexpressing *edeB* can significantly improve edeine production in *B. brevis*, which may have wide applications in medicine and agriculture.

## Conclusion

In this study, we identified and characterized a ParB family transcription factor, EdeB, which significantly increased the expression of several genes on *ede* BGC, and significantly improved the production of edeine A and B. In addition, our results demonstrated that EdeB directly bound to the promoter region of *ede* BGC to improve the transcription and translation of edeine genes. Our study elucidated the molecular mechanism underlying the biosynthesis of edeines, and can be used to improve the production of edeines in industry in the future.

## Data availability statement

The data presented in the study are deposited in the NCBI repository, accession number NZ_CP023474.1 and ATF13884.1.

## Author contributions

JD, QLi and WC designed the study; JD, CZ, LZ and QLo conducted the experiments. JD and LZ performed data collection and analyses. JD, QLi and WC drafted the manuscript. All authors have read, edited, and approved the final manuscript.

## Funding

This study was supported by the National Natural Science Foundation of China (31772216, 32000047, and 31501698), the Science and Technology Project of Hunan Province, China (2021NK1040), and High-tech Industry Science and Technology Innovation Leading Project of Hunan province, China (2020NK2006).

## Conflict of interest

The authors declare that the research was conducted in the absence of any commercial or financial relationships that could be construed as a potential conflict of interest.

## Publisher’s note

All claims expressed in this article are solely those of the authors and do not necessarily represent those of their affiliated organizations, or those of the publisher, the editors and the reviewers. Any product that may be evaluated in this article, or claim that may be made by its manufacturer, is not guaranteed or endorsed by the publisher.
