# Supplementary material for: Characterization of a pathway−specific activator of edeine biosynthesis and improved edeine production by its overexpression in Brevibacillus brevis
Source: Front Plant Sci. 2022 Oct 25;13:1022476. doi: 10.3389/fpls.2022.1022476 (PMC9641203; doi:10.3389/fpls.2022.1022476)
Supplement: Supplementary file 1 [file Table_1.docx]

Supplementary Material

Supplementary Table 1 The primers used in this study

| Primer names | Sequence (5′→3′) |
| --- | --- |
| pE194-F | CAGCTGCCTCGCGCGTTTCGGTGA |
| pE194-R | GTTAAGGGATGCATAAACTGCATC |
| edeB-F1 | AGGCACACGAAAAACAAGTTAAGGGATGCAGTTTATGCATCCCTTAACTACGATAAGCAGGGTGGGATG |
| edeB-F2 | TTCAAATTGCAGATCATAACTTCGTATAATGTATGCTATACGAACGGTAAGATTTGCCAGCACATCGTA |
| edeB-R1 | GTCGATTGGCTGAATAACTTCGTATAGCATACATTATACGAACGGTAGTATCGTATCGGAAGGGCATGT |
| edeB-R2 | TGTGTCAGAGGTTTTCACCGTCATCACCGAAACGCGCGAGGCAGCTGCGATATTCCGGTGCATCATACA |
| edeB-Px-F | CGCGGATCCAATATGTACGATGTGCTGGCAAATCT |
| edeB-Px-R | CATGCCTGCAGTTACGCATCGCACATATACACG |
| Apra-F | TACCGTTCGTATAGCATACATTATACGAAGTTATGATCTGCAATTTGAATAATAACC |
| Apra-R | TACCGTTCGTATAATGTATGCTATACGAAGTTATTCAGCCAATCGACTGGCGAGCG |
| edeA-RT-F | ATTCTTCTCCGCGATTTTCAG |
| edeA-RT-R | CGTTCCCGTTTCATCCACTT |
| edeQ-RT-F | GCGATATTGGCTGGAGCATAA |
| edeQ-RT-R | CATGAGGACACGATGGTTGG |
| edeK-RT-F | TTCCTGGTGAGACGATTGAGTT |
| edeK-RT-R | TTCCGACAACGACATCCTCTT |
| 16s-RT-F | ATGCGTAGAGATGTGGAGGAA |
| 16S-RT-R | GCGGAGTGCTTATTGCGTTA |
| P1 | TAGCCAAGCCAAATGTTGT |
| P2 | GTTGAGAAGCTGACCGATGAG |
| P3 | AGGAAGGTCCAGTCGGTCAT |
| P4 | CAGGAGCTACCGAACGAT |
| xylR-5-out | GGGATTTTGGTCATGAGATTA |
| pAD123-end | GCCATTGGGATATATCAACGGT |
| edeB- F | CCCCTCTAGAAATAATTTTGTTTAACTTTAAGAAGGAGATATACCATGTACGATGTGCTGGCAAATCTA |
| edeB-R | GGGCTTTGTTAGCAGCCGGATCTCAGTGGTGGTGGTGGTGGTGCTCGAGTCGCATCGCACATATACACG |
| edePro-F | GCTTCTTATCCTGATGCAATGC |
| edePro-R | TCTAGCGCTGCATAGAATGG |
